# Mechanical analyses of critical surgical maneuvers in the correction of cleft lip nasal deformity

**DOI:** 10.1371/journal.pone.0195583

**Published:** 2018-04-13

**Authors:** Hanyao Huang, Yeping Li, Xiangyou Luo, Xu Cheng, Bing Shi, Jingtao Li

**Affiliations:** State Key Laboratory of Oral Diseases & National Clinical Research Center for Oral Diseases & Dept. of Oral Maxillofacial Surgery, West China Hospital of Stomatology, Sichuan University, Chengdu, China; New York University Langone Medical Center, UNITED STATES

## Abstract

The relapse of nasal deformity is a challenge for modern correction of cleft lip. A comprehensive understanding in the biomechanical perspective of both the formation and correction of the cleft lip nasal deformity would lead to improved stability of the corrective outcome. In this study, a finite element model of secondary cleft lip nasal deformity was constructed, on which two critical corrective maneuvers were mimicked in the form of force-loading. The intercrural suture was simulated by a force loaded at the intermediate crus of the alar cartilage directing anteriorly and medially, and the suture suspending the alar cartilage to the upper lateral cartilage was simulated by a force loaded at the lateral crus directing superiorly and medially. The equivalent von-mises stress and the total deformation consequent to different patterns of loading were captured. Our biomechanical analyses suggested that the intercrural suture at the nasal tip might be more effective in generating widespread morphological change than the suspension suture, but left much higher level of stress within the skin envelope if placed too high. Synergistic effect was observed between the two sutures in both the resultant deformation and stress. In addition, our simulations were partially supported by clinical photogrammetry data.

## Introduction

Cleft lip deformity is one of the most common congenital anomalies in humans. After centuries of evolution, modern corrective procedures could well restore the morphology of the lip, but the residue asymmetry and deviation in the nose is still a significant challenge to plastic surgeons. Among patients with cleft lip, the alar dome on the cleft side is collapsed and the alar base and nasal tip deviated laterally. Although satisfactory nose morphology could be achieved immediately after corrective procedures, the relapse of the deformity would almost always occur in varying degree.

The shape of the nose is decided by its underlying cartilage framework. In the deformity of cleft lip nose, the alar cartilage is hypoplasic and displaced caudally and posteriorly, and thus leads the collapse and deviation of its covering soft tissue [1–4]. During the corrective procedure, the hypoplastic alar cartilage would be enhanced with various grafts and restored to normal position, but all of these maneuvers would leave tension within the soft tissue, which is considered the major cause of replase. For decades, cleft surgeons have been trying to acquire insights into the biomechanics of cleft lip nasal deformity [5]. It was assumed that detailed analyses and documentation of the biomechanical consequence of different surgical maneuvers would serve as a strong predictor for the stability of the corrective outcomes or the occurrence of relapse.

Finite element modeling has been validated as a helpful biomechanic tool in the study of rhinoplasty. By dividing the complex structure of nose into a collection of subdomains, it can efficiently specify regions with high levels of distortion or stress. For example, finite element analysis (FEA) has been successfully applied in the analyses of the formation of the inverted-V deformity [6], the effectiveness of the septal L-strut [7, 8], and the form of the nasal tip [9–12]. In the field of cleft lip and palate, FEA has been employed in analyzing the labial musculature [13] and the maxillary bone [14–16], but its advantage has not been sufficiently taken in the study of cleft lip nasal deformity.

In this study, a finite element model was established basing on the regional MRI scanning of a patient with unilateral cleft lip nasal deformity, with both the cartilage framework and the skin envelope constructed. Two critical surgical maneuvers were analyzed: the suture suspending the lower alar cartilage on the cleft side to the upper lateral cartilage, and the suture fixing the medial crura of bilateral alar cartilage at the nasal tip. Each sutures were mimicked by loading the model with corresponding forces, and the biomenchanical consequences in both the stress distribution and morphology changes documented in details.

## Materials and methods

### MRI imaging

A 25-year-old Chinese Han male patient with secondary unilateral cleft lip nasal deformity was enrolled in November, 2016. Clinical examination revealed typical asymmetry and collapse in the outer nose of cleft lip nose of the volunteer. MRI scanning was performed for the nasal region in the Department of Radiology, West China Hospital of Sichuan University. According to our preliminary experiments, t1-mpr-ns-say-p2-iso, 4’18”; t2-spc-ns-say-p2-iso, 4’00” was chosen as the MRI scanning sequences. DICOM-format images were exported into Mimics 15.0 (Mimics, Materialise, Belgium) for reconstruction.

### Nasal model construction

**Fig 1:**
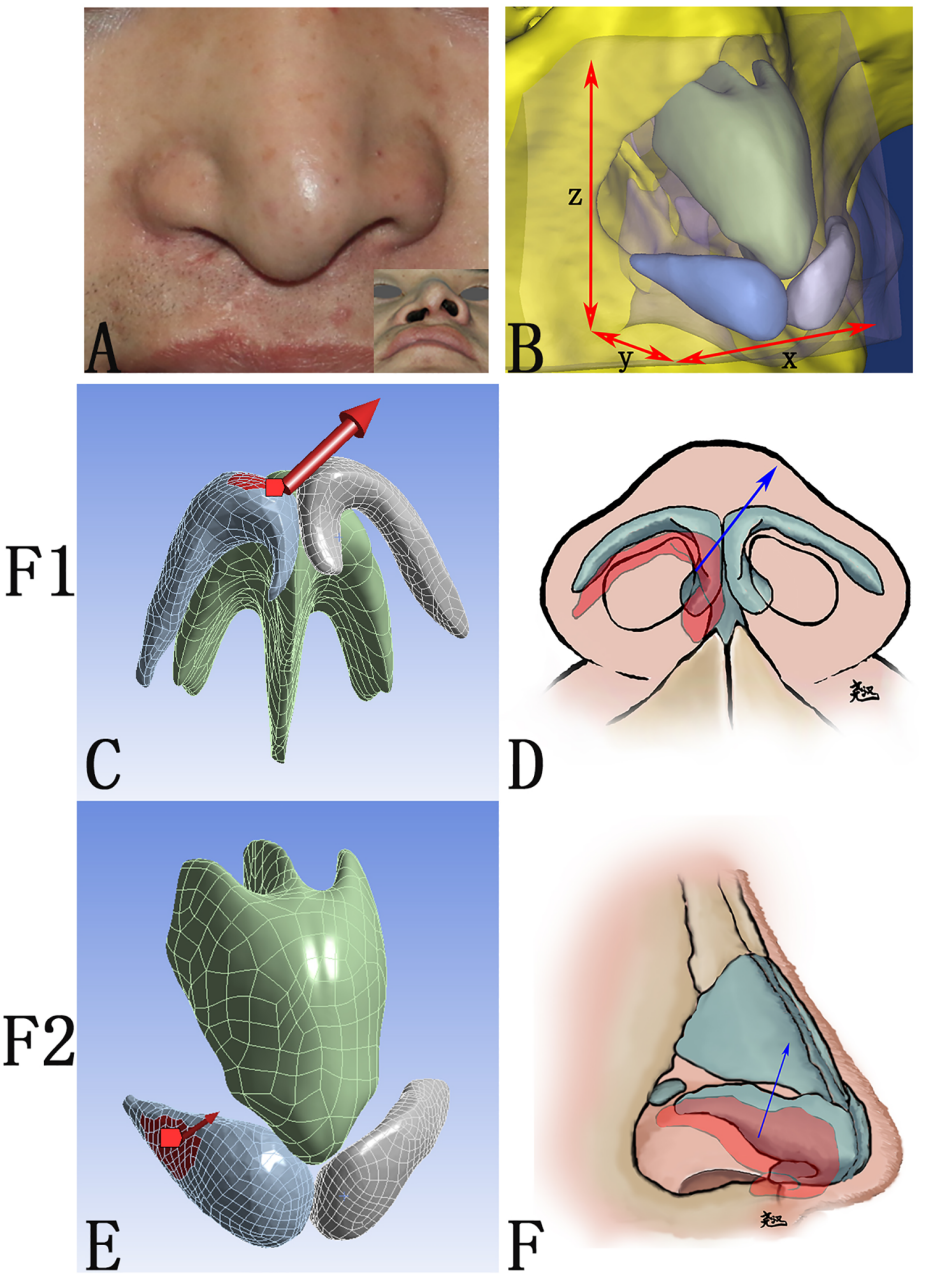
The CAD model for finite element analysis and directions of forces for CAD model. (A) A 25-year-old Chinese Han male patient with secondary unilateral cleft lip nasal deformity. (B) Width (x), thickness (y) and height (z) of the CAD model, composed of the nasal framework and the skin envelope. (C, D) The intercrural suture was simulated by a force vector (F1) which was set at the margin of the intermediate crus of the alar cartilage directing anteriorly and medially. (E, F) The suspension suture was simulated by a force vector (F2) set at the lateral crus of the alar cartilage directing superiorly and medially.

The nasal model was constructed according to the MRI data and general anatomy of cleft lip nasal deformity. The three dimensional model was composed of two parts, the nasal framework and the skin envelope. The nasal framework consisted of two alar cartilages and the T-bar-shaped cartilaginous complex including the septal cartilage and the upper lateral cartilages (Fig 1A). The cartilage framework and the skin envelope were assembled through Pro/Engineer 5.0 (Pro/E) (PTC, Needham, MA, USA). The dimensions, including width, thickness, height and volume were shown in Table 1. Physical properties of the cartilages and the skin envelope were assigned according to published data as shown in Table 2 [6]. Finally, mesh generation of the model was performed using Workbench 15.0 (ANSYS Inc., Canonsburg, PA, USA) and exported in ASM format [17]. The definition of the meshwork was shown in details in Table 3.

**Table 1:** Definition of models for finite element analysis.

Geometry	Length X(x; mm)	Length Y(y; mm)	Length Z(z; mm)	Volume(v; mm^3^)
The skin envelope	57.03	69.93	45.92	94277.00
The left alar cartilage	13.83	15.29	12.02	388.50
The right alar cartilage	13.78	17.62	10.98	494.06
The T-bar-shaped cartilaginous complex	18.08	24.97	25.49	2646.60

**Table 2:** Elastic properties of materials in the model for finite element analysis.

Materials [6]	Young’s modulus (MPa)	Density (kg/m^3^)	Poisson ratio
The skin envelope	0.5	980	0.33
The nasal cartilage	0.8	1080	0.15

**Table 3:** Definition of FE model in ANSYS Workbench.

Geometry	The skin envelope	The left alar cartilage	The right alar cartilage	The T-bar-shaped cartilaginous complex	total
Nodes	39088	4460	5571	6297	50363
Elements	24025	2385	3085	3438	29554

### Finite element simulation

The peripheral margin of the skin envelope and the bony structure was set fixed and the cranial margin of the cartilage framework was set attached to the piriform aperture to emulate the connection between the upper cartilage and the maxilla. Forces of two vectors were loaded to simulate the surgical maneuvers during unilateral cleft lip nose correction. The intercrural suture was simulated by a force vector (F1) which was loaded at the margin of the intermediate crus of the alar cartilage directing anteriorly and medially (Fig 1B and 1C); The suspension suture was simulated by a force vector (F2) loaded at the lateral crus of the alar cartilage directing superiorly and medially (Fig 1D and 1E). Various magnitudes of forces were applied in different simulations: 1. F1 alone at 5N; 2. F2 alone at 5N; 3. F1 and F2 loaded at the same time and both at 5N; 4. F2 alone at 15N; 5. F2 alone at 5N with bilateral alar cartilages merged together. Static structural analysis was applied to calculate the total deformation (TD) and the equivalent von-mises stress (EQV) during the simulations.

### Photogrammetry

A total of forty-two patients who had received secondary cleft rhinoplasty combining suspension suture and passive intercrural suture, together with forty healthy age-matched volunteers, were enrolled in this research. All patients were of non-syndromic unilateral cleft nose deformity. Photogrammetric analyses were performed using Image-pro plus 6.0 (Media Cybernetics, Inc., Maryland, USA), and all measurements were listed in Table 4 and described in S1 Fig and S1 Table. The basal-view photos were used for analyses. Each photograph was measured three times by the second author with an interval of 4 weeks between measurements. Independent samples t-test were performed to compare postoperative and healthy measurements. Paired samples t-test was applied to compare preoperative and postoperative measurements. All statistical analyses were operated with SPSS software version 17.0. Statistical significance for all linear and angular measurements was determined at p-value <0.05.

**Table 4:** Statistical analyses of measured parameters among three groups.

Parameters#	Preoperative Group	Postoperative Group	Paired Samples Test	Postoperative Group	Normal Group	Independent Samples Test
			P-value*			P-value*
Deviation of nasal tip	0.8792±0.10	0.9192±0.066	ΨΨ	0.9192±0.066	0.9645±0.031	ΨΨ
The convex contour of alar lobule	146.47±7.25	137.41±7.37	ΨΨ	137.41±7.37	131.75±7.48	Ψ
The distance relationship of columella between the two sides	0.5779±0.13	0.8256±0.18	ΨΨ	0.8256±0.18	0.9537±0.029	ΨΨ

*The threshold of significance was set at P = 0.05. “Ψ” means P<0.05; “ΨΨ” means P<0.001.

^#^ Parameters: Deviation of nasal tip: The absolute distance ratio of nasal tip between Prn-Ipr and Prn-Ipl; The convex contour of alar lobule: The convex angle of alar lobule between Prn-Sf line and Ac-Sf line; The distance relationship of columella between the two sides: The vertical distance ratio of nasal columella between bilateral Nt to the Sn horizontal line. The definitions of anthropometric landmarks were demonstrated in S1 Fig.

The research protocol was censored and approved by the Ethic Committee of West China Hospital of Stomatology, Sichuan University (Approval No.WCHSIRB-D-2016-084R1). Individual participants could not be identified during or after data collection. Written informed consents were acquired from all the subjects enrolled in this study (as outlined in PLOS consent form).

## Results

The TD was scaled in millimeter and demonstrated in color coded arrows on the nasal cartilage framework and contour bands on the skin envelope. Stress distribution was measured in EQV scalar value which combined the normal and shear stresses for each element under a complex 3D loading condition.

**Fig 2:**
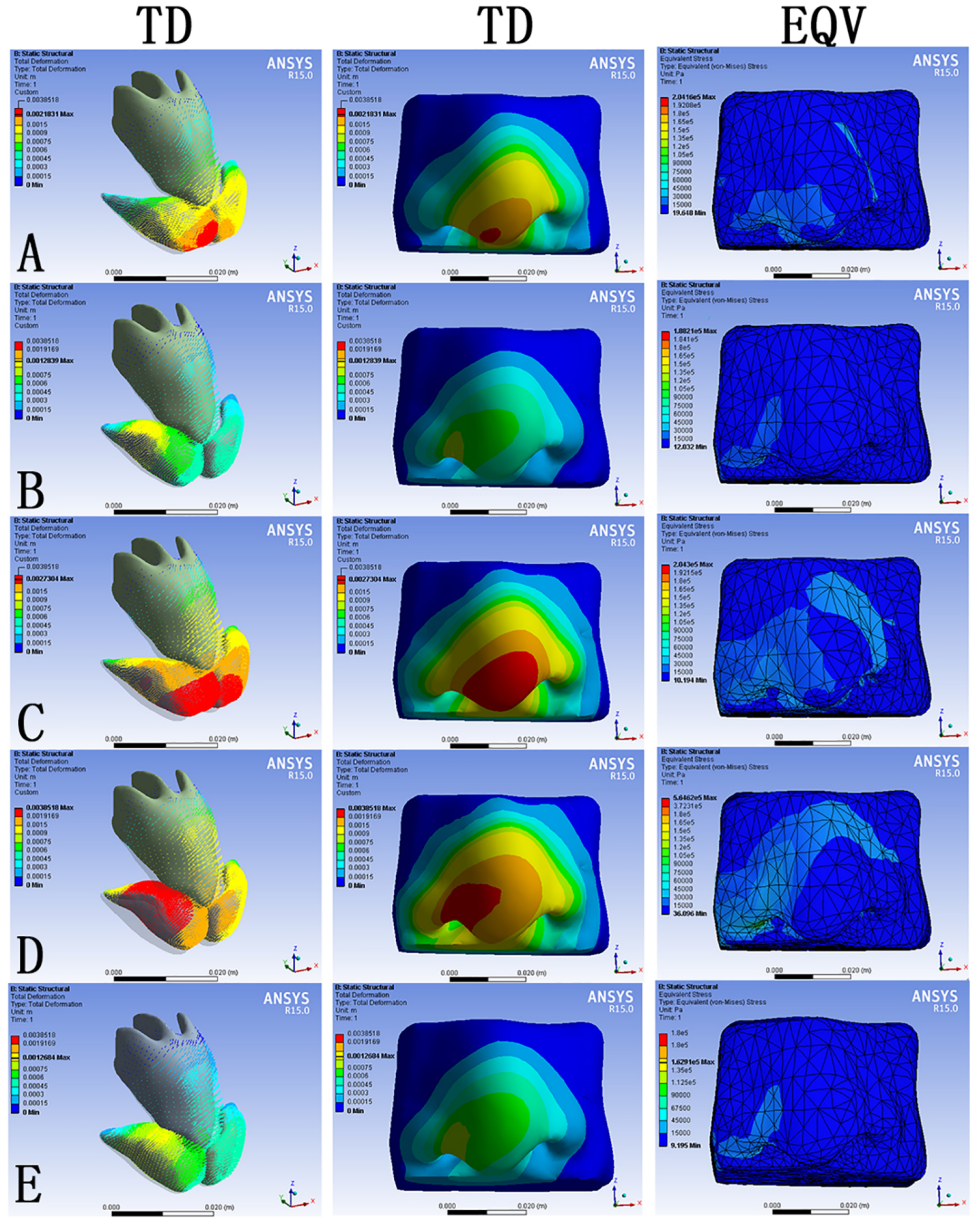
The TD on the nsaal framework and the TD and the EQV on the skin envelope. (A) F1 alone at 5N. (B) F2 alone at 5N. (C) F1 and F2 both at 5N. (D) F2 alone at 15N. (E) F2 alone at 5N with bilateral alar cartilages merged together. Blue indicated the stable part as control. The changes of TD (mm) and EQV (kPa) were corresponding to color change.

When the model was loaded with F1 alone at 5N, the maximum deformation on the cartilage framework was observed at the margin of the loaded intermediate crus of the alar cartilage near the nasal tip, extending to the ipsilateral medial crus, the ipsilateral lateral crus, the T-bar-shaped cartilaginous complex, and the contralateral alar cartilage (Fig 2A, left). On the skin envelope, the maximum deformation was observed at the nasal tip, extending all the way to the ipsilateral alar base, the columella base, the nasal radix and the contralateral alar base (Fig 2A, middle). The EQV concentrated on the alar dome, the nasal tip, the ipsilateral alar base and the ipsilateral columella base, affecting the contralateral dorsum (Fig 2A, right).

When the model was loaded with F2 alone at 5N, the maximum deformation on the cartilage framework was observed at the margin on the lateral crus near the dome, affecting the ipsilateral intermediate crus, the ipsilateral medial crus and the neighboring upper lateral cartilage, extending to the contralateral alar cartilage (Fig 2B, left). The deformation around the ipsilateral dome on the skin envelope was slightly larger than other regions. The TD, when compared with F1 alone, was more evenly distributed in the triangle region between bilateral alar bases and the nasal radix (Fig 2B, middle). The EQV concentrated on the alar dome and the alar groove on the loaded side (Fig 2B, right).

When the model was loaded with F1 and F2 at the same time both at 5N, the maximum deformation on the cartilage framework was observed at the bilateral intermediate crura and the loading region of F2, extending to the ipsilateral medial crus, the neighboring upper cartilage and the contralateral alar cartilage (Fig 2C, left). On the skin envelope, the maximum deformation was observed at the nasal tip, extending all the way to the ipsilateral alar base, the nasal radix and the contralateral alar base, with gradual attenuation (Fig 2C, middle). The EQV, similar to loading of F1 alone at 5N, was distributed around the dome of alar, the nasal tip, the ipsilateral alar base and the ipsilateral columella base, affecting the contralateral dorsum (Fig 2C, right).

Since the simulated suspension suture (F2 alone) also demonstrated contour changes in the nasal tip, though at a smaller scale when compared with the simulated intercrural suture, we wondered if F2 at greater magnitude could result in greater tip projection. In the next simulation, F2 alone at 15N was loaded. The maximum deformation on the cartilage framework was observed at the margin on the lateral crus near the dome, affecting the ipsilateral intermediate crus, the ipsilateral medial crus and the neighboring upper lateral cartilage, extending to the contralateral alar cartilage, with a higher value of TD than loading F2 alone at 5N (Fig 2D, left). On the skin envelope, the distribution of TD was similar to that when loading F2 alone at 5N, but more widespread. The deformation around the nasal tip, however, was approximate to that when loading F1 alone at 5N (Fig 2D, middle). The EQV, which was obviously higher than loading F2 alone at 5N, concentrated on the ipsilateral dome and the alar base, extending all the way along the ipsilateral nasal wall to the nasal radix and affecting the contralateral dorsum (Fig 2D, right).

Then we further examined if the effect of the suspension suture (F2) could be strengthened by stabilizing the medial crura of bilateral alar cartilages. The bilateral alar cartilages were connected at the medial crus into a single element, and F2 at 5N was loaded. The deformation pattern and stress distribution were similar to the simulation without merging bilateral medial crura (Fig 2E).

**Fig 3:**
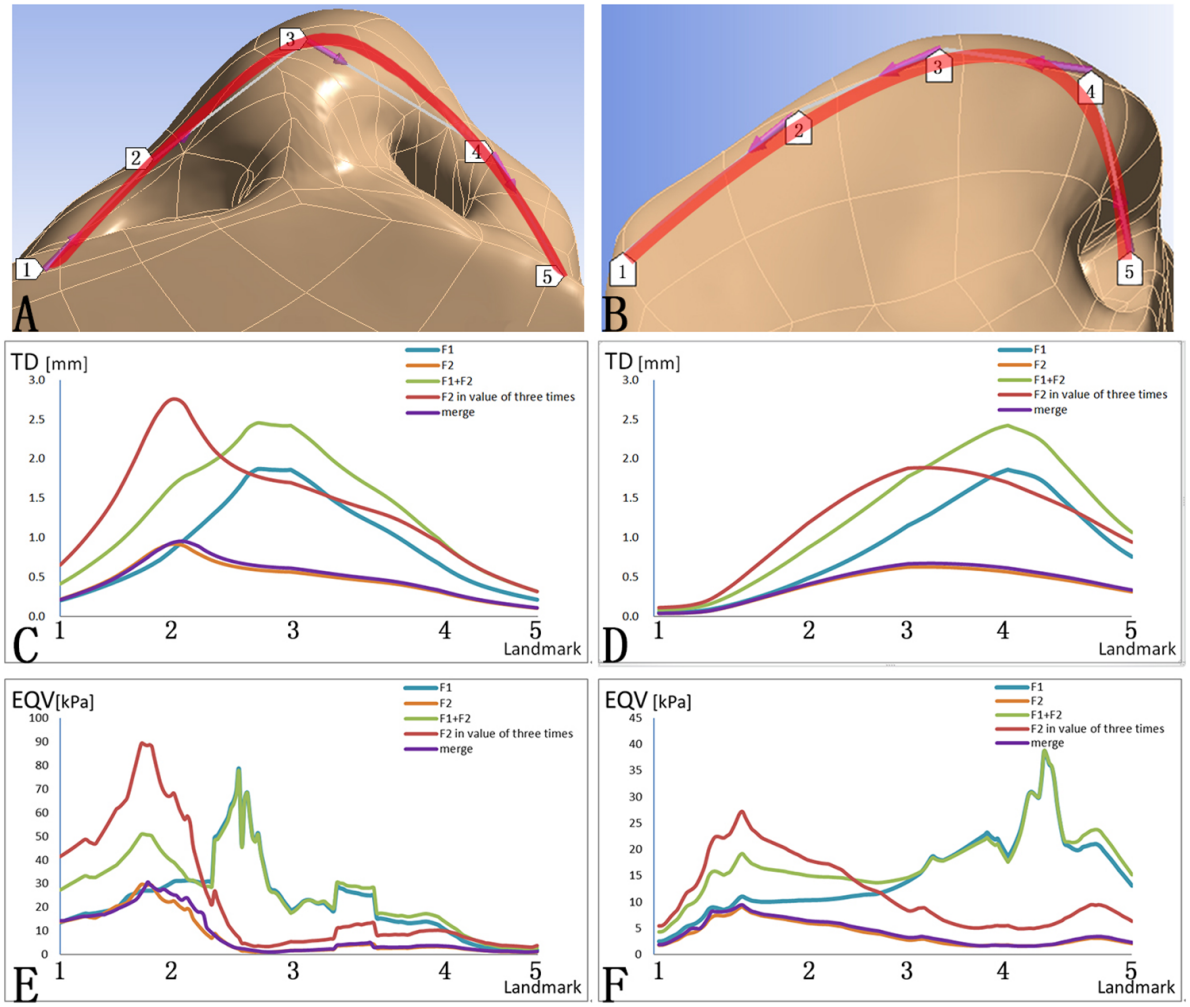
The TD and EQV on two paths on the skin envelope. (A) Path one started from one alar base through the nasal tip to the alar base at the other side. Path one started from landmark one to landmark five: Landmark one represented the alar base at the cleft side; Landmark two represented the dome at the cleft side; Landmark three represented the nasal tip; Landmark four represented the dome at the non-cleft side; Landmark five represented the alar base at the non-cleft side. (B) Path two started from the columella base through the nasal tip to the nasal radix. Path two started from landmark one to landmark five: Landmark one represented the nasal radix; Landmark three represented the dorsum; Landmark four represented the nasal tip; Landmark five represented the columella base; Landmark two was the midpoint of the neighboring two landmarks. (C) The TD on Path one. (D) The TD on Path two. (E) The EQV on Path one. (F) The EQV on Path two. Lines of five colors represented five ways of force loading.

Two paths on the cutaneous surface were defined to specify the TD and EQV at critical nasal landmarks. Path one was defined by the alar bases at both sides (landmarks one, five), the alar domes at both sides (landmark two, four) and the nasal tip (landmark three) (Fig 3A). Path two was defined by the nasal radix (landmark one), the dorsum (landmark two), the midpoint of the dorsum and the nasal tip (landmark three), the nasal tip (landmark four) and the columella base (landmark five) (Fig 3B). When set at the same magnitude, F2 generated significantly smaller TD than F1 from the dome to the contralateral alar base on Path one and all the way on Path two (Fig 3C and 3D, Blue, Orange). When loading F1 and F2 at the same time, the TD was similar to the superposition of those generated by F1 and F2 alone (Fig 3C and 3D, Blue, Orange, Green). When loading F2 at 15N, the resultant TD around the nasal tip was almost even to that generated by loading F1 alone (Fig 3C and 3D, Blue, Red). When set at the same magnitude, F2 generated significantly lower EQV than F1 on Path one and two (Fig 3E and 3F, Blue, Orange). When loading F1 and F2 at the same time, the EQV was higher from the alar base at the cleft side to the ipsilateral dome of alar, and almost even to loading F1 alone from the ipsilateral dome to the contralateral alar base on Path one (Fig 3E, Blue, Green), and the EQV on Path two was higher from the nasal radix to the midpoiont between the dorsum and the nasal tip (Fig 3F, Blue, Green). On path one, F2 in value of three times resulted significantly higher EQV than F1 from the alar base at the cleft side to the ipsilateral dome. On path two, F2 in value of three times resulted significantly higher EQV than F1 from the nasal radix to the dorsum (Fig 3E and 3F, Blue, Red). The merge of bilateral alar cartilages introduced no notable influence on either the TD or EQV generated by F2 loading (Fig 3C, 3D, 3E and 3F, Orange, Purple).

Detailed results of clinical photogrammetry were shown in Table 4.

## Discussion

Nasal deformities among patients with cleft lip have always been challenging to plastic surgeons. Typical characteristics of unilateral cleft lip nasal deformities include collapsed alar dome and deviated nasal tip and columella. Besides intrinsic tissue hypoplasia on the cleft side, the majority of cleft nose deformities originates from displacement of the underlying cartilage framework [18–21]. Generally, the affected alar cartilage is flattened and downwardly displaced. During surgical correction, traction forces generated by sutures could well restore the position of the cartilage and thus the normal contour of the nose, but the frequent relapse of the deformity remains unsolved.

The causes of the relapse, as generally recognized, rest in the postoperative tension within the tissue. Given with time, even small magnitude of tension could drag the alar cartilage from the corrected position to its origin one, and thus results in deformity relapse during the healing process. All these factors justify the importance of overcorrection for both primary and secondary surgery. From this mechanical perspective, a thorough understanding of the stress distribution within the nose generated by surgical correction would contribute to improved surgical technique and stable long-term outcome. With the advancement of computational technology and finite element analysis, detailed biomechanical analysis of nasal structures could be performed under simulated circumstances. Finite element modeling has been successfully applied in the studies of nasal structure to further our understanding in the mechanical aspects of nasal tip projection [9–12], alar cartilages positioning [22], and the support of nasal septum L-strut [7, 8, 23–26] and various nasal implants [27]. MRI was chosen to reveal the probable location of the cartilages and offered an accurate morphology of the cartilage framework to provide the basis for further modeling.

Two frequently used surgical maneuvers were simulated on our model: (1) the medial fixation called the intercrural suture of the medial crus was mimicked by a medially directed force (Fig 1B and 1C) and (2) the superior suspension of the lateral crus was mimicked by a superiorly directed force (Fig 1D and 1E). The deformation pattern predicted the immediate surgical outcome and the distribution and magnitude of stress, in certain degree, suggested the probability of relapse.

**Fig 4:**
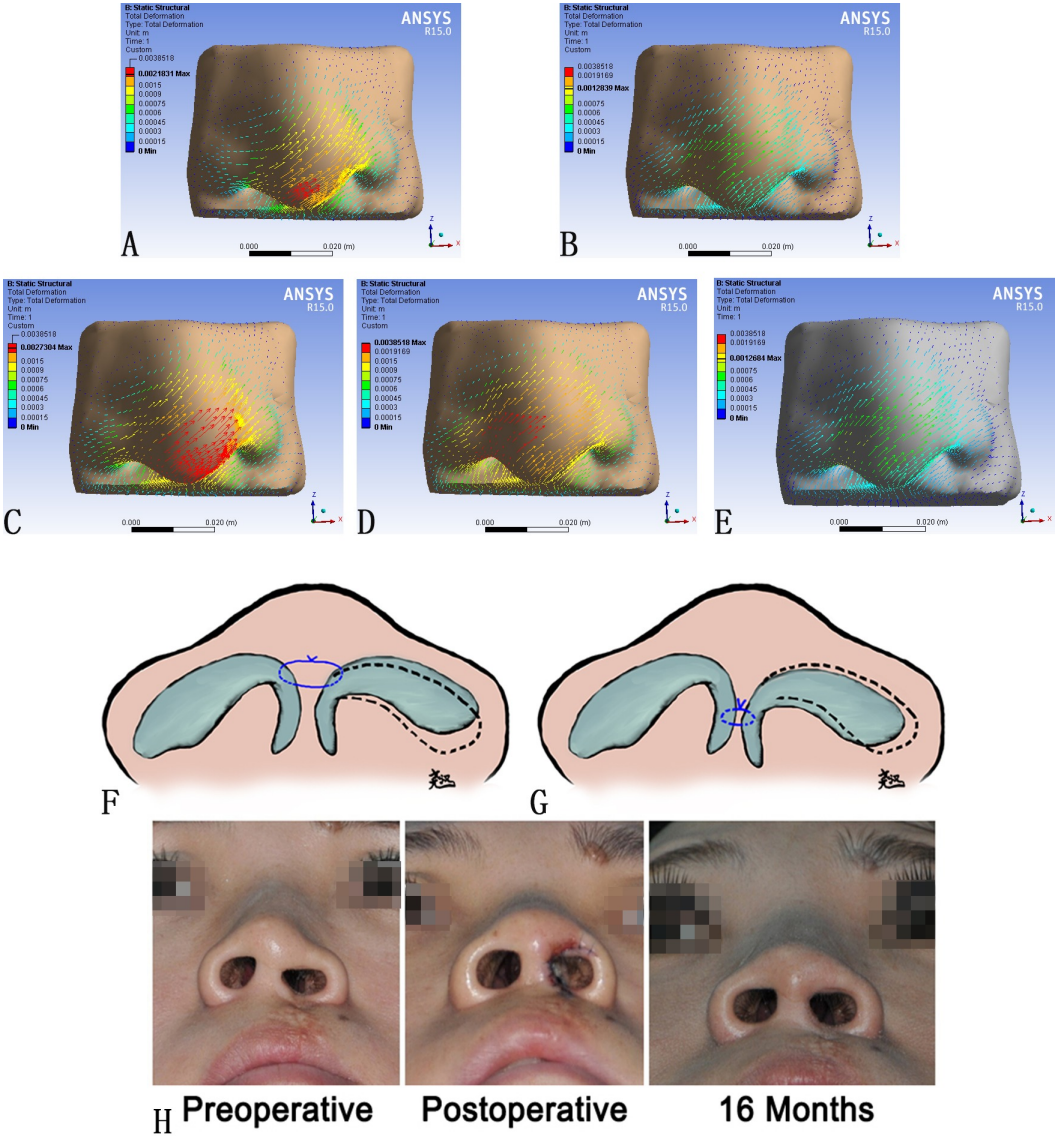
The vectors of deformation under different types of force loading and the Schematic drawing for this operative technique. (A) TD with F1 alone at 5N. (B) F2 alone at 5N. (C) TD with F1 and F2 both at 5N. (D) TD with F2 alone at 15N. (E) TD with F2 alone at 5N with bilateral alar cartilages merged together. The change of TD (m) was corresponding to color change. The length of the arrow represented the value of deformation, and the direction of the arrow represented the direction of deformation. To distinguish (B) and (E), the skin envelope was set in grey when loading F2 alone after merging two alar cartilages. (F, G) Schematic drawing of two different places on the medial crus to coapt the alar cartilages together in basal view: 1. High position of suture on the medial crus (F); 2. Low position of suture on the medial crus (G). (H) A representative case of unilateral cleft nose deformity repaired with the suspension suture and the passive intercrural suture.

When set at the same magnitude, F1 led to more widespread deformity than F2 (Figs 2A, 2B, 4A and 4B). The deformation generated by F1 was more notable around the nasal tip than the alar, which was consistent with clinical observation (Fig 4A). The deformation generated by F2, on the other hand, focused more on the alar region, especially the alar dome (Fig 4B). The maximum stress generated by F1 was observed around the dome and columella, while that generated by F2 was observed around the dome and alar base. These data suggested that the suture at the medial crus might be more efficient in restoring the nasal symmetry but with greater stress within the tissue.

Synergistic effect was observed between F1 and F2 (Fig 3). The deformation caused by loading F1 and F2 at the same time was almost the superposition of those caused by loading F1 and F2 respectively. The stress, similarly, also increased significantly. Therefore, it indicated that although multiple sutures could result in more significant change in the nasal morphology, but also left greater amount of stress within the tissue which might lead to higher risk of relapse.

Different forces might lead to similar morphological changes. When F2 was set at a magnitude three times of that of F1, the deformation in the sagittal direction at the nasal tip was identical to that caused by F1 (Fig 3), but three-time F2 failed to recapitulate the narrowing of the nasal tip in the horizontal direction as generated by F1. Also, the stress generated by three-time F2 was even higher than that generated by loading F1 and F2 at the same time, further suggesting that the intercrural could not be totally replaced by the suspension suture.

In our simulation, F1 was designed to represent the effect of intercrural suture at the nasal tip. When F1 was exerted on top of F2, the stress increased significantly (Fig 2C). In contrast, passive connection between the bilateral medial crus did not increase the stress generated by F2 (Fig 2E). Such an observation suggested that although the intercrural suture was effective in enhancing the tip projection, it was at the cost of significant increase in stress. In cases where a pointy nasal tip was not demanded, an oriental nose for example, placing the intercrural suture at a lower position might reduce the risk of relapse (Fig 4F and 4G).

Photogrammetry was performed to verify this hypothesis acquired from FEM study. According to the measurements, significant improvements were observed in the collapse deformity of the alar on the cleft side (Preoperative group = 146.47 degrees; Postoperative Group = 137.41 degrees, p<0.05), nasal tip deviation (Postoperative group = 0.9192±0.066; Normal group = 0.9645±0.031, p<0.05), columella shortage (Postoperative group = 0.8256±0.18; Normal group = 0.9537±0.029, p<0.05) and alar collapse (Postoperative group = 137.41±7.37; Normal group = 131.75±7.48, p<0.05). Moreover, long-term outcomes revealed minimal relapse (Fig 4H).

Finite element analysis was a purely theoretical research technique with its intrinsic limitations. All mechanical properties of the model were set in an elastic region, which could be both elastic and plastic in reality [6]. The real properties of human tissue are heterogeneous, which would definitely influence the mechanical results. Also, the magnitude of the forces generated by sutures could not be accurately measured and were loaded with presumed approximates. The loading spot and direction of the force were difficult to set in accuracy and were chosen basing on experience. In addition, we have to confess that multiple features importance to the long-term stability of rhinoplasty was not fully recapitulated in this model due to technical limitations. First, the upper and lower lateral cartilages were set as separated so as to mimic the suspension suture that moved the lower lateral cartilage close to the upper, which could not be recapitulated if they were set as a continuous complex. Second, the dissection between the overlying skin and the cartilages rendered the model too complex for accurate calculation that due to technical difficulties. Third, scar contracture, which was a crucial influential factor for postoperative stability, was impossible to mimic for the uncertainty of either the magnitude or direction of the contractile forces.

## Conclusion

In this finite element study, two operative maneuvers in correcting cleft lip nasal deformity were recapitulated: the suture connecting the medial crura of bilateral alar cartilage, and the suture suspending the alar cartilage to the upper lateral cartilage. The mimicked intercrural suture at the nasal tip was more effective in generating widespread morphological change when compared with the suspension suture, but left much higher level of stress in the skin envelope if placed too high. Synergistic effect was observed between the two sutures in both the resultant deformation and stress.

## Supporting information

S1 Fig Photogrammetric measurements of the innovation parameters. From top to bottom on the photograph the parameters are: Parameter A-deviation of nasal tip: The absolute distance ratio of nasal tip between Prn-Ipr and Prn-Ipl; Parameter B-the convex contour of alar lobule:The convex angle of alar lobule between Prn-Sf line and Ac-Sf line; Parameter C-the distance relationship of columella between the two sides: The vertical distance ratio of nasal columella between bilateral Nt to the Sn horizontal line.
(PDF)

S1 Table Definitions of anthropometric landmarks. (PDF)
