# Supplementary figures and images for: Mechanical analyses of critical surgical maneuvers in the correction of cleft lip nasal deformity
Source: PLoS One. 2018 Apr 13;13(4):e0195583. doi: 10.1371/journal.pone.0195583 (PMC5898757; doi:10.1371/journal.pone.0195583)

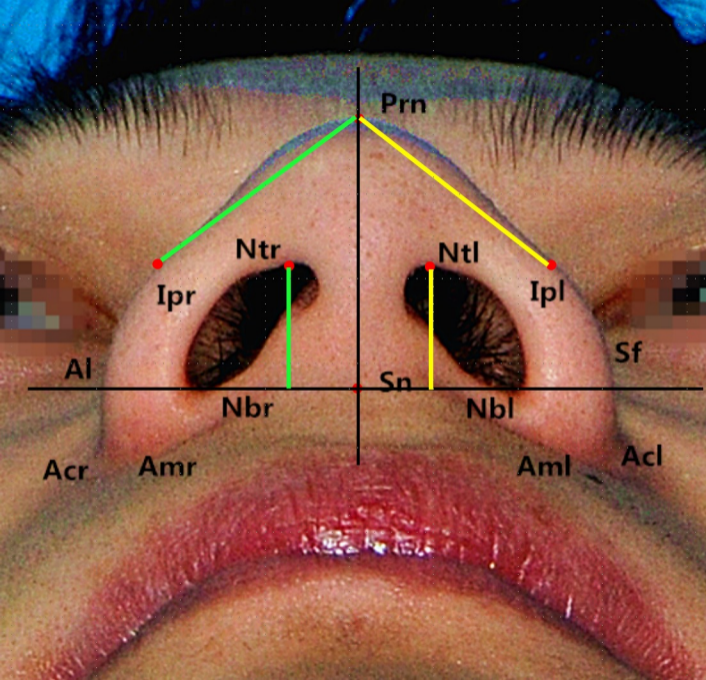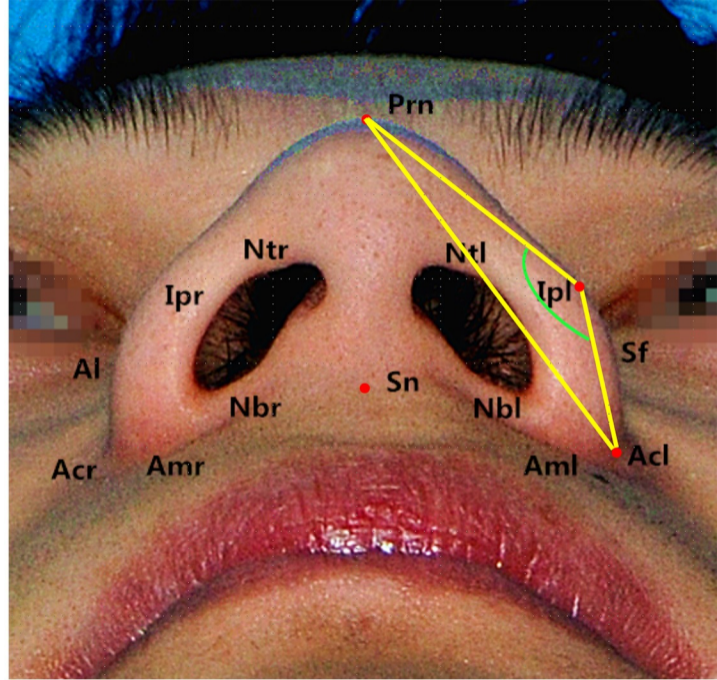

Supplement: S1 Fig — From top to bottom on the photograph the parameters are: Parameter A-deviation of nasal tip: The absolute distance ratio of nasal tip between Prn-Ipr and Prn-Ipl; Parameter B-the convex contour of alar lobule:The convex angle of alar lobule between Prn-Sf line and Ac-Sf line; Parameter C-the distance relationship of columella between the two sides: The vertical distance ratio of nasal columella between bilateral Nt to the Sn horizontal line. (PDF) [file pone.0195583.s001.pdf]
