# Supplementary material for: Mechanical analyses of critical surgical maneuvers in the correction of cleft lip nasal deformity
Source: PLoS One. 2018 Apr 13;13(4):e0195583. doi: 10.1371/journal.pone.0195583 (PMC5898757; doi:10.1371/journal.pone.0195583)
Supplement: S1 Table — (PDF) [file pone.0195583.s002.pdf]

S1 Table. Definitions of Anthropometric Landmarks

Landmarks	Definitions
Prn	The most anterior point of the nose
Ntr and Ntl	The top point of the nostril
Acr and Acl	The most lateral point in the curved base line of each alar
Sn	The midpoint of the nasolabial angle at the columella base
Ipr and Ipl	The intersection point between higher Nt horizontal line and bilateral nose contour
Sfr and Sfl	The maximum vertical distance point of alar contour to the ipsilateral Prn-Ac line
